# Validation of a Novel Collection Device for Non-Invasive Urine Sampling from Free-Ranging Animals

**DOI:** 10.1371/journal.pone.0142051

**Published:** 2015-11-04

**Authors:** Lisa Michelle Danish, Michael Heistermann, Muhammad Agil, Antje Engelhardt

**Affiliations:** 1 German Primate Center, Junior Research Group Sexual Selection, Göttingen, Germany; 2 Endocrinology Laboratory, German Primate Centre, Göttingen, Germany; 3 Faculty of Veterinary Medicine, Bogor Agricultural University, Bogor, Indonesia; Colorado State University, College of Veterinary Medicine and Biomedical Sciences, UNITED STATES

## Abstract

Recent advances in non-invasively collected samples have opened up new and exciting opportunities for wildlife research. Different types of samples, however, involve different limitations and certain physiological markers (e.g., C-peptide, oxytocin) can only be reliably measured from urine. Common collection methods for urine to date work best for arboreal animals and large volumes of urine. Sufficient recovery of urine is thus still difficult for wildlife biologists, particularly for terrestrial and small bodied animals. We tested three collection devices (two commercially available saliva swabs, Salivette synthetic and cotton, and cotton First aid swabs) against a control to permit the collection of small volumes of urine from the ground. We collected urine samples from captive and wild macaques, and humans, measured volume recovery, and analyzed concentrates of selected physiological markers (creatinine, C-peptide, and neopterin). The Salivette synthetic device was superior to the two alternative devices. Concentrations of creatinine, absolute C-peptide, C-peptide per creatinine, absolute neopterin, and neopterin per creatinine measured in samples collected with this device did not differ significantly from the control and were also strongly correlated to it. Fluid recovery was also best for this device. The least suitable device is the First aid collection device; we found that while absolute C-peptide and C-peptide per creatinine concentrations did not differ significantly from the control, creatinine concentrations were significantly lower than the control. In addition, these concentrations were either not or weakly correlated to the control. The Salivette cotton device provided intermediate results, although these concentrations were strongly correlated to the control. Salivette synthetic swabs seem to be useful devices for the collection of small amounts of urine from the ground destined for the assessment of physiological parameters. They thus provide new opportunities for field studies to incorporate physiological markers, particularly on smaller bodied and terrestrial animals and where urine collection is difficult.

## Introduction

Recent advances in non-invasively collected samples, such as faeces, have opened up new and exciting opportunities for wildlife research encompassing a range of disciplines such as conservation genetics, field endocrinology, behavioural ecology and so forth, covering topics ranging from costs of behavioral strategies to reproductive biology and individual health [1–5]. In addition to faeces, physiological field studies nowadays use samples such as hair, feathers, saliva, and urine [6,7], depending on the parameter to be measured and what is possible to be collected. Different types of samples, however, involve different limitations, including species specific storage effects and variable hormone excretion lag times [5]. Certain physiological markers (e.g., C-peptide, oxytocin), for example, can only be reliably measured from urine [5, 8–9]. Such sample collection by field biologists is regularly accomplished non-invasively, with no need to capture or even interfere with the animal’s natural behaviour [1–7, 10–20]. In many studies on wildlife, animals are habituated to observers and thus allow individual identification. In others, individual identification is not necessary. Common collection methods for urine, however, work best for arboreal animals as they rely on catching streams of urine [5, 13–14]. Only in a few cases is it possible to pipette urine directly from the ground (e.g., [15]) since it requires a sufficient amount of urine and a ground that does not allow fast seepage of fluids. Urine may also be recovered by centrifuging urine soaked soil (e.g., [16]), although this requires an electric centrifuge and becomes more difficult as urine is more widely dispersed on the ground. While urine has been successfully collected from snow (e.g., [17,18]), the excess water may reduce the concentration of the marker of interest below the detection ability of the assay, particularly for smaller bodied animals. Recovery of urine from terrestrial animals is thus currently still difficult at best, and may not yield sufficient volume for certain measures. Even the collection of urine from semi-terrestrial and arboreal species may be challenging, since the urine stream usually dissipates so that collection requires rather large devices like umbrellas,big plastic tarps or bags (e.g., 100x50cm plastic sheet) [14, 19–20]. Collectors also need to be fast to successfully place the device under the respective animal. Often enough, urine remains to be pipetted from leaves [14–15, 19], though dispersion can make pipetting difficult. The use of unusual collection methods indicates the extent of the difficulties faced by researchers; for example, Monfort (2003) reported that he was only able to collect urine from wild mongooses after he inadvertently discovered that they would urinate on rubber sandals. Sufficient recovery of urine is thus still difficult for wildlife biologists, and developments of new collection devices are urgently needed.

When developing new collection devices, it has to be taken into account that materials used during collection and storage of biological samples can influence marker concentration [8, 21]. For example, it has been shown that salivary assays for testosterone, DHEA, progesterone, and estradiol result in artificially raised concentrations for thee markers [21]. In addition, C-peptide concentration was decreased for urine samples that were absorbed into filter paper, then reconstituted [8]. Any novel collection device or repurposed device must therefore be thoroughly validated.

The aim of our study was to find a suitable collection device to use for urine collection from various animal species, including terrestrial ones, and to validate it for different physiological markers, i.e. urinary C-peptide, neopterin and creatinine. C-peptide is created when insulin (the hormone mediating metabolic activity) is formed from proinsulin and thus provides an indication of body condition and energetic status [22]. It is nowadays increasingly used in primatological field studies [13, 15, 23]. Neopterin, a pteridine, is released by activated monocytes/macrophages as part of cellular immune activation [24]; since a wide variety of disease conditions in which the cell mediated immune system is activated result in an increase in this marker, it provides a broad indication of health [24–26]. Creatinine is a break-down product of creatine phosphate in muscle. Since it is produced at a fairly constant rate, it is typically used to control for differences in urine concentration and to standardize the concentration of analytes measured from urine [27].

We tested a range of potential collection devices including sterile first aid swabs, since these are easy to acquire in the field. A disadvantage to these swabs is that there is no immediate recovery device to facilitate recovery from the swab via centrifugation. Given this issue, we additionally tested devices provided for the collection of saliva samples, because we assumed that these devices may also be useful for the collection of small amounts of urine. Amongst these potential collection devices are Salivettes from Sarstedt. These come in three varieties: 1) Salivette saliva examination with a 100% cotton swab; 2) Salivette Cortisol code blue with a synthetic swab; and 3) Salivette citric acid. We chose to test only the first two, since the citric acid in the third device is just an additive to induce saliva production and thus of no use for collecting urine. The first Salivette is a simple cotton swab, whereas the second one has specifically been developed to increase recovery of cortisol from small volume samples. Since there is always a risk of substance loss due to surface absorption with any absorbent material, the material of this swab has been particularly designed to catch cortisol in small volumes and/or of low concentrations. We therefore intended to test whether, in addition to cortisol, it also collects other substances better than cotton.

Our first specific aim was to examine differences in volume recovery in the different collection devices. For this, we determined volume recovery rate using water. Since materials used during collection and storage of samples can influence marker concentration [8, 21], we then examined for all three devices the recovery rate of urinary C-peptides. For the analysis, we collected urine samples from humans and captive macaques. In order to test whether the collection device proving best in this analysis is in general a good device for collection of urine, even when analyzing other markers, we additionally examined urinary neopterin from samples collected with this specific device. Following laboratory analyses, our last specific aim was to evaluate the applicability of the best collection device to field collection. For this, we collected urine samples with the specific device from wild crested macaques living in a lowland rainforest habitat in northern Sulawesi, Indonesia [28] and checked whether the volume gathered would have been enough for subsequent analysis. Much of the ground of lowland rainforest is covered with leaf litter, which makes it difficult to recover urine with conventional methods. Furthermore, in our study area, ground consists mainly of sand, which allows fast seepage of fluids. It is therefore an ideal test ground for urine collection devices to be used under challenging conditions.

## Materials and Methods

### Ethics statement

All research undertaken strictly adhered to all animal care, legal and ethical requirements of Germany and Indonesia, as well as the Animal Behavior Society (ABS) and Association for the Study of Animal Behaviour (ASAB) "Guidelines for the Use of Animals in Research", the American Society of Primatologists (ASP) "Principles for the Ethical Treatment of Non-Human Primates" and the recommendations of the Weatherall report “The use of nonhuman primates in research”.

The German Primate Center is registered and authorised by the local and regional veterinary governmental authorities (Reference number: 32.22/VO Stadt Göttingen; 392001/7 Stadt Göttingen). All experiments were performed in accordance with the German Animal Welfare Act (“Tierschutzgesetz der Bundesrepublik Deutschland”). This includes supervising and advice by the institutional animal welfare officer and the institutional animal welfare body (“DPZ Tierschutzkommission”; approval number E2-15).

All macaque urine samples for this study were collected non-invasively (i.e., animals were not handled at all; see below). Human samples were voluntarily collected from the authors (LMD; MH, AE) of this paper and a colleague. All urine sample donors gave their verbal consent to use their urine for the purpose of the study. All samples were analysed anonymously and the data published do not allow drawing any inference about the identity of the donor.

### Collection devices

We tested the following three collection devices: 1) a 100% sterile cotton swab from a first aid kit purchased in Indonesia (hereafter First aid); 2) Salivette saliva examination with a 100% cotton swab from Sarstedt (order number 51.1534) (hereafter Salivette cotton); and 3) Salivette Cortisol code blue with a cylindrical swab made of synthetic material from Sarstedt (order number 51.1534.500) (hereafter Salivette synthetic). Both Sarstedt collection devices include both a swab and a tube; the tube has a removable component that fits the swab, with a small hole in the bottom to allow extraction of liquids using a centrifuge. We used tubes provided with the Salivette collection devices to extract urine from the first aid swabs.

### Quantifying volume recovery from collection devices in the lab

We quantified volume recovery by pipetting 500μL of water onto the different collection devices (four samples each). We also cut First aid collection devices in half since they are large. We measured the volume recovered by determining the mass of water recovered and calculating the volume using density. For fluid recovery, in a first step, we used a manually operated centrifuge for one minute. We then placed all samples in an electric centrifuge (3,000 rpm, five minutes) to see if additional volume could be recovered.

### Determination of C-peptide recovery

#### Sample collection

For the determination of C-peptide recovery, we opted to collect urine from both healthy human and nonhuman primate subjects so that there would be variation in C-peptide concentration due to body size differences [29]. We collected one sample each from four humans, three rhesus macaques, and one long-tailed macaque. Human samples were collected in collection jars and pipetted into smaller tubes. Nonhuman primate samples were collected from healthy macaques at the German Primate Center. The animals are kept under conditions documented in the European Directive 2010/63/EU (directive on the protection of animals used for experimental and other scientific purposes) and the EU Recommendations 2007/526/EG (guidelines for the accommodation and care of animals used for experimental and other scientific purposes). These conditions are consistent with the regulations of the Guide for Care and Use of Laboratory Animals by the National Research Council (USA). Specifically, all macaques were housed indoor in standardized cages under daily surveillance by veterinarians and animal caretakers. The monkeys were kept with a 12:12 light-dark schedule at a temperature between 18–23°C and a humidity range of 50–60%. All nonhuman primate rooms are provided with 8–10 fresh air changes per hour. Animals were fed twice a day with commercial monkey chow supplemented with fruits and vegetables. Water was available *ad libitum*. Environmental enrichment was provided by placing rings and perches into the cages, foraging or task-oriented feeding methods (e.g. treats, vegetables or fruits frozen in ice cubes, food puzzle), and playing music.

All samples were collected noninvasively by pipetting urine from plastic mats placed under each cage, and only samples uncontaminated with feces were collected. All samples were frozen at -20°C until transferal to collection devices.

#### Transferal of samples to the collection devices

All urine samples (n = 32) were first thawed and brought to room temperature. For each sample, an aliquot of 500 μL of urine was pipetted onto each of the collection devices, and then placed into a Salivette tube in a Styrofoam cooler with ice to replicate field conditions. An additional aliquot of 500 μL of each sample was kept as control placed in a refrigerator (2–8°C). First aid collection devices were placed into empty Salivette tubes, since these are designed for liquid recovery from swabs. Since samples collected in the field often remain on ice for the remainder of the observation day, we left the cooler with the samples in Salivettes in a dark place, at room temperature, for seven hours. This allowed us to test the effect in field conditions and with the samples in the collection device for several hours. We then left the samples in the Salivettes at room temperature for 30 minutes prior to recovering the urine using a manually operated centrifuge. All recovered samples and the control samples were then frozen at -20°C until analysis. Since freezing and thawing can affect marker concentrations, we ensured that all samples (including controls) were treated identically in this regard.

#### C-peptide analysis

C-peptide concentrations were measured using a commercial C-peptide ELISA Kit from IBL International GmbH, Hamburg, Germany (Art. No. RE 53011) designed for the measurement of C-Peptide in human and which has been validated for the measurement of C-peptide concentrations in macaque urine [8, 14]. Prior to C-peptide assay, human urine samples were diluted 1:20 and macaque samples were diluted 1:2 with IBL sample diluent (Art. No. RE 53017) to ensure that concentrations of C-peptides would be within the detection range of the assay. We then assayed 100 μL of the diluted urine using the protocol provided by the manufacturer. As usual, all 32 samples from the 4 conditions were run in duplicate on a single assay plate since sample number was lower than the maximum capacity of one plate [8,9,13,14,15,19,23,25]. Since all sample duplicate values were within the range of 7% [25], no samples had to be re-measured. Assay sensitivity was 0.064 ng/mL. Intra-assay coefficients of variation calculated from the measurement of low, middle, and high value quality controls run with the assay were all below 5%. Since only a single plate was run (see above), no inter-assay (i.e. between-plate) variation is reported. Previous studies showed that inter-assay variation for the C-peptide assay used is <15% [8,14].

To account for differences in urine concentration, C-peptide values were adjusted to urinary creatinine (measured using the method by Bahr et al. 2000). Prior to creatinine measurement, each sample was first diluted 1:10 with distilled water. Intra-assay coefficients of variation calculated from the measurement of low and high value quality controls run with the assay were both below 5%. Again, since all samples were measured on a single plate, inter-assay coefficients of variation are not reported. C-peptide concentrations are presented as ng C-peptide/mg creatinine, though we also present analyses for creatinine and absolute C-peptide concentrations, i.e. levels expressed per mL of urine.

### Determination of neopterin recovery

#### Sample collection

Based on the results of the C-peptide analysis, we only tested the Salivette synthetic collection device for recovery of neopterin. Here we made use of urine samples collected from twelve healthy rhesus macaques (6 females, 6 males) at the German Primate Center as part of a different study evaluating the suitability of urinary neopterin measurements under field conditions (Heistermann and Higham in prep.; approval number E1-15). Animals were housed as described above and sample collection and transferal was conducted in the same way as described above.

#### Neopterin analysis

Samples were analyzed after being diluted 1:10 to 1:200 (depending on concentration) with assay buffer using a commercial enzyme-immunoassay kit (ELISA Kit from IBL International GmbH, Hamburg, Germany, Art. No. RE 59321). As with the C-peptide analysis (see above), all samples (n = 24) were measured in duplicate on a single assay plate. All duplicate values were within 7% of each other and thus no sample measurement had to be repeated (see above). The detection limit of the assay was 0.18 ng/mL. Intra-assay coefficients of variation calculated from the measurement of low, middle, and high value quality controls run with the assay were all below 5%. Again, since all samples were measured on a single assay plate, inter-assay coefficients of variation are not reported (see above). To account for differences in urine concentration, neopterin values were adjusted to urinary creatinine measured as described above. Final neopterin concentrations are presented as ng neopterin/mg creatinine, though we also present analyses for creatinine and absolute neopterin concentrations, i.e. levels expressed per mL of urine.

### Field test of urine volume recovery

The field component of the study was conducted as part of the Macaca Nigra Project in the Tangkoko-Batuangus Nature Reserve in northern Sulawesi (1°34’N, 125°14’E). We opportunistically collected urine from adult male crested macaques (n = 11) using the Salivette synthetic collection device in March 2014. All adult individuals were individually recognizable from unique characteristics of their callosities, crest, scars, etc. Individuals were also habituated to human observers, allowing close observation (approximately 5m). We were thus able to observe urination. We waited for the urine to fall onto a substrate, e.g. leaf litter, and the animal to leave if on the ground before pressing the collection device into the urine. The material of the collection device highly facilitates urine absorption. Urine was extracted from the collection devices at the end of the observation day using a manually operated centrifuge for two minutes at high speed. We estimated approximate volume recovered using a dropper, which indicated volumes of 0.5 and 1.0 mL.

### Data analysis

We used a Friedman test and subsequently the Wilcoxon exact test to test for significant differences in the concentration of absolute C-peptide, creatinine, and C-peptide per creatinine levels obtained from each collection device and the control. The Wilcoxon exact test was used since our samples size was below the threshold to determine p-values accurately using asymptotic tests (n = 8). For neopterin, we only ran a Wilcoxon exact test (since n = 12) for absolute neopterin, creatinine, and neopterin per creatinine concentrations. All analyses were two tailed since we had no a priori prediction regarding direction of any differences between the treatments. We then further examined our data to determine if marker concentrations from urine from each collection device were correlated to control concentration since the previous test determines if there are overall treatment differences and we also wanted to know if the concentration of the test condition was dependent on the control. All data were checked for normality using a Shapiro-Wilks test. Since concentrations of C-peptide per creatinine and neopterin per creatinine were normally distributed, we determined Pearson’s correlation coefficients for these markers, but Spearman’s correlation coefficients for absolute C-peptide, creatinine, and absolute neopterin. All statistics were run using the statistical software R v2.1.5.1 [30]; we used packages stats (Friedman and Shapiro-Wilks tests), and Hmisc (correlations) and package exactRankTests for the Wilcoxon exact tests.

To determine if enough urine was collected in the field for analysis, we assumed that 200 μL or more was enough for analysis, 150–199 μL was uncertain (since some volume may be lost during storage), and less than 150 μL was insufficient (based on the laboratory procedure for measuring C-peptides; see [14,15]).

## Results

We were able to recover, 39.7% (standard deviation 1.6%), 60.1% (6.6), 33.3% (1.3), and 84.5% (1.9) of the water from manual centrifugation from the First aid, First aid half, Salivette cotton and Salivette synthetic collection devices respectively. These percentages increased to 64.5% (0.8), 76.0% (5.6), 55.4% (1.1), and 92.2% (1.1) after the use of the electric centrifuge.

Creatinine concentrations differed significantly between the control and samples collected in the collection devices (Friedman test, χ^2^ = 19.75, df = 3, p < 0.001; Table 1) in that creatinine concentrations were significantly lower than the control when using Salivette cotton and First aid collection devices (Wilcoxon tests, V = 36, p = 0.008; V = 45, p = 0.004). The recorded differences were small though, i.e. not exceeding 10%. Creatinine concentrations from samples collected in Salivette synthetic collection devices, however, did not differ significantly from the control (Wilcoxon test, V = 8, p = 0.375). The concentrations of creatinine for all collection devices were significantly correlated with the controls (Salivette synthetic: r = 0.98, p < 0.001; Salivette cotton: r = 1.00, p < 0.001; First aid: r = 1.00, p < 0.001). Results were equivalent for creatinine concentration from samples used in the neopterin test; there was no significant difference in creatinine concentration between the Salivette synthetic collection device and the control (Wilcoxon test, V = 15, p = 0.124) (Table 2), and the concentration of creatinine from the Salivette synthetic collection device was significantly correlated with the control (r = 1.00, p < 0.001).

**Table 1:** Effect of different collection devices on urinary C-peptide and creatinine concentrations. Concentrations are reported as percent of control, mean and SD.

Collection Device	C-peptide concentration (absolute)	Correlation with control	Creatinine concentration	Correlation with control	C-peptide concentration (per creatinine)	Correlation with control
First aid	104.0 ± 33.4	r = 0.83, p< 0.001	92.0 ± 3.5*	r = 1.00, p<0.001	112.9 ± 35.0	r = 0.67, p = 0.071
Salivette cotton	113.8 ± 19.7*	r = 0.98, p<0.001	90.0 ± 7.1*	r = 1.00, p<0.001	128.9 ± 25.1*	r = 0.97, p<0.001
Salivette synthetic	89.2 ± 13.7	r = 0.98, p<0.001	102.8 ± 5.5	r = 0.98, p<0.001	87.0 ± 15.8	r = 0.93, p<0.001

* Concentrations that significantly differ from the control based on Wilcoxon signed ranks tests.

**Table 2:** Effect of Salivette synthetic devices on urinary neopterin and creatinine concentrations. Concentrations are reported as percent of control, mean and SD.

Collection Device	Neopterin concentration (absolute)	Correlation with control	Creatinine concentration	Correlation with control	Neopterin concentration (per creatinine)	Correlation
Salivette synthetic	99.4 ± 12.3*	r = 0.99, p<0.001	102.9 ± 6.8*	r = 1.00, p<0.001	97.4 ± 12.9*	r = 0.96, p<0.001

* Concentrations that significantly differ from the control based on Wilcoxon tests.

Absolute C-peptide concentrations differed significantly between the control and samples collected in the different devices (Friedman test, χ^2^ = 12.80, df = 3, p = 0.005) (Table 1), in that the C-peptide concentrations from Salivette cotton collection devices were significantly higher than the control (Wilcoxon test, V = 0, p = 0.016). Absolute C-peptide concentrations from samples collected in Salivette synthetic and First aid collection devices, however, did not significantly differ from the control (Wilcoxon tests, Salivette synthetic: V = 30, p = 0.109; First aid: V = 13, p = 0.547). For all collection devices, the concentrations of absolute C-peptide were significantly correlated with the controls (Salivette synthetic: r = 0.98, p < 0.001; Salivette cotton: r = 0.98; p < 0.001, First aid: r = 0.83, p < 0.001).

**Fig 1:**
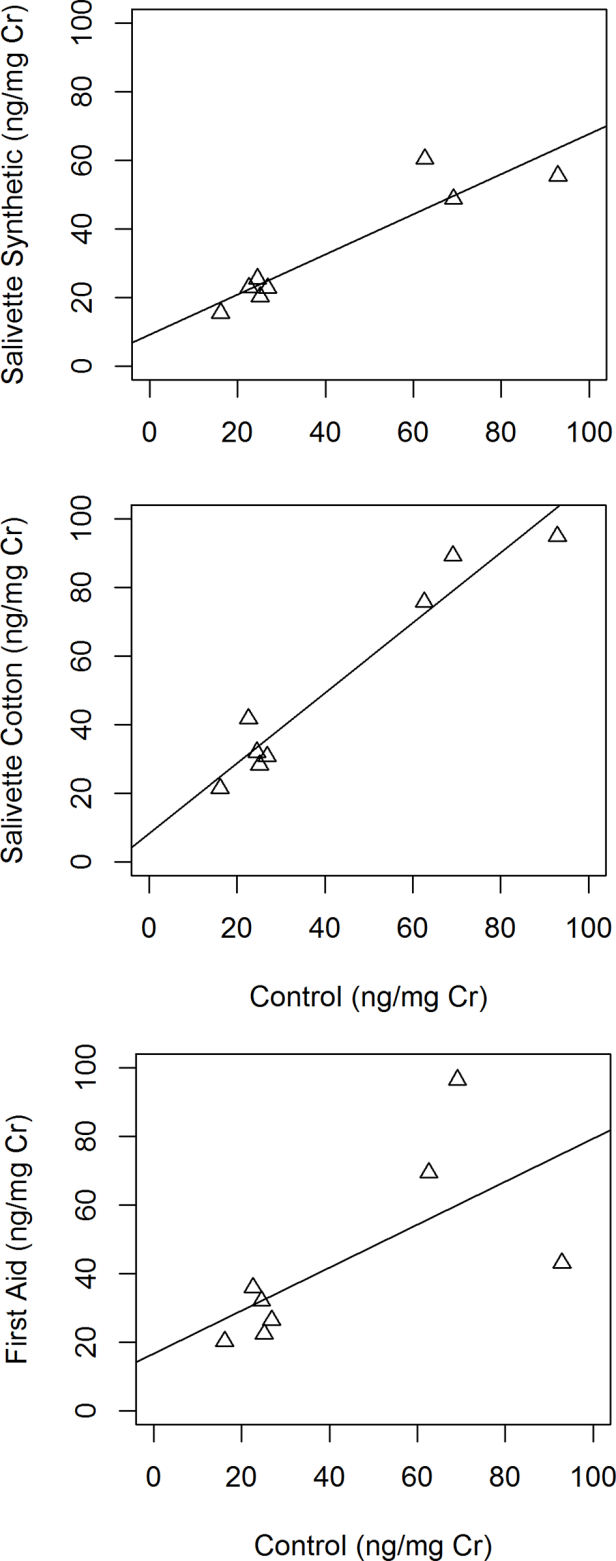
a) Correlation between urinary c-peptide per creatinine concentrations measured from controls and after recovery from Salivette synthetic collection devices (Pearson correlation coefficient, rs = 0.93, p<0.001). b)Correlation between urinary c-peptide per creatinine concentrations measured from controls and after recovery from Salivette cotton collection devices (Pearson correlation coefficient, rs = 0.97, p<0.001). c) Correlation between urinary c-peptide per creatinine concentrations measured from controls and after recovery from First aid collection devices (Pearson correlation coefficient, rs = 0.67, p = 0.071).

C-peptide per creatinine concentrations differed significantly between the control and samples collected in the collection devices (Friedman test, χ^2^ = 14.55, df = 3, p = 0.002) (Table 1), in that the C-peptide per creatinine concentration was significantly higher using the Salivette cotton collection device compared to the control (Wilcoxon test, V = 0, p = 0.008). C-peptide per creatinine concentration from the Salivette synthetic and First aid collection devices, however, did not differ significantly from the control (Wilcoxon tests,Salivette synthetic: V = 32, p = 0.055; First aid: V = 11, p = 0.383). The concentration of C-peptides per creatinine were significantly correlated with the controls for the Salivette synthetic (r = 0.93, p < 0.001, Fig 1A) and Salivette cotton collection devices (r = 0.97, p < 0.001, Fig 1B). The concentration of C-peptides per creatinine were not significantly correlated with the controls for the First aid collection device (r = 0.67, p = 0.071, Fig 1C).

Absolute neopterin concentrations did not differ significantly between the Salivette synthetic collection device and the control (Wilcoxon test, V = 39, p = 0.624). The concentrations of absolute neopterin were also significantly correlated with the control (r = 0.99, p < 0.001).

**Fig 2:**
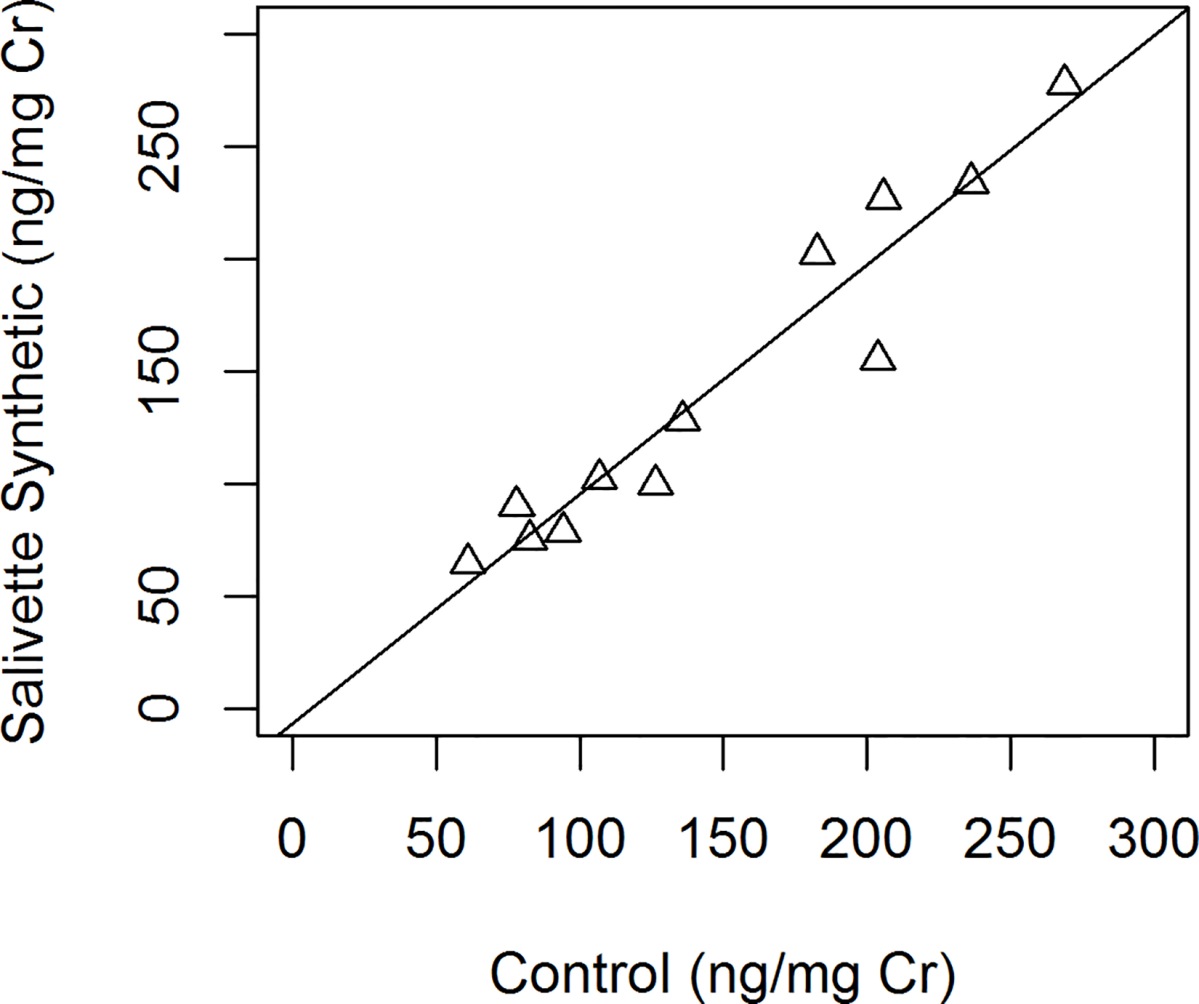
Correlation between urinary neopterin per creatinine concentration measured from controls and after recovery from Salivette synthetic collection device (Pearson correlation coefficient, r = 0.96, p<0.001).

Neopterin per creatinine concentrations did not differ significantly between the Salivette synthetic collection device and the control (Wilcoxon test, V = 43, p = 0.791) (Table 2). The concentration of neopterin per creatinine was also significantly correlated with the control (r = 0.96, p < 0.001, Fig 2).

We were able to recover urine from all collection devices in the field (Table 3). Overall, 54.5% of samples collected were of sufficient volume for analysis, with an additional 18.2% possibly being of sufficient volume. Moreover, we were able to accurately qualitatively assess whether enough urine could be collected prior to collection.

**Table 3:** Urine volume recovery in the field.

Sample no.	Estimated Volume (mL) Recovered	Enough for Analysis?
1	0.00	No
2	0.10	No
3	0.10	No
4	0.15	Maybe
5	0.15	Maybe
6	0.20	Yes
7	0.35	Yes
8	0.70	Yes
9	0.75	Yes
10	0.90	Yes
11	0.95	Yes

## Discussion

Our results show that amongst our tested collection devices, Salivette Cortisol code blue with a synthetic swab is superior to the two alternative devices tested for the collection of urine samples. This device seems to be particularly suitable for the collection of urine when sample volume and/or marker concentration is expected to be small, as demonstrated by the clearly highest fluid recovery rate of almost 85% when using a manually operated centrifuge. Moreover, concentrations of creatinine, absolute C-peptide, C-peptide per creatinine, absolute neopterin, and neopterin per creatinine measured in samples collected with this device did not differ significantly from the control; concentrations were also strongly correlated to the control. Notably variation in creatinine concentrations in all experimental treatments were within the normal assay variation range. Recovery of fluid was high and clearly best for the Salivette synthetic collection device, using both a manually operated and electric centrifuge; best volume recovery was, however, obtained with an electric centrifuge. Moreover, good urine recovery was possible in the field using the Salivette synthetic collection device. We therefore recommend the use of the Salivette synthetic device, as it is suitable for obtaining both absolute and relative measures of markers and has an excellent recovery rate.

Based on our results, the least suitable device is the First aid collection device. For this, we found that while absolute C-peptide and C-peptide per creatinine concentrations did not differ significantly from the control, creatinine concentrations were significantly lower than the control. Since creatinine is an important control measure for urine concentration, this deficit potentially impacts the interpretation of results of any marker analysis based on urine samples from wild animals. While concentrations of creatinine were significantly correlated with the control, concentrations of absolute C-peptide were significant, but weakly correlated with the control and there was no significant correlation between concentrations of C-peptide per creatinine, likely as a result of the high variance in this measure. Therefore, we consider the First aid collection device unsuitable for urine sample collection due to the high variance in C-peptide concentrations and corresponding differences from control.

The Salivette saliva collection device provided intermediate results. Here, some of the recovery differed from the control; creatinine concentrations were significantly lower, and absolute C-peptide and C-peptide per creatinine concentrations significantly higher than the control. However, concentrations were strongly correlated to the control, indicating this device is suitable for relative comparisons of C-peptide concentration, but not absolute. Notably, since this collection device artificially inflates the concentration of C-peptides, it may be useful for smaller bodied species, whose normal C-peptide concentrations are low and thus difficult to measure [29]. The poor fluid recovery rate of this device, which is likely due to the high absorbency of the cotton material, however, may negate this potential advantage, depending on the volume of urine possible to collect.

In summary, the device designed for increasing the recovery of cortisol from saliva samples produced similarly good recovery rates for the three markers examined in our study deriving from urine samples. We thus assume that devices designed for the collection of cortisol in saliva are in general very good urine collection devices also for other physiological markers. It is our hope that our validation of these salivary cortisol collection devices for the collection of two additional markers will thus provide new opportunities for field studies, particularly on smaller bodied animals and where urine collection is difficult. Urine collection using this method is feasible for free-ranging animals that can be observed at a close distance. Depending on the research question, individual identification may or may not be required. Notably, individual identification can be accomplished through a variety of means (i.e., physical traits, color banding) and has been successful on a variety of taxa [1–7, 10–20]. We nevertheless recommend investigators to carry out preliminary field tests on urine volume recovery for their specific study species and to consider electric centrifuges where possible.

## Supporting Information

S1 Table Data for C-peptide Analysis. (DOCX)

S2 Table Data for Neopterin Analysis. (DOCX)
