# Supplementary material for: Validation of a Novel Collection Device for Non-Invasive Urine Sampling from Free-Ranging Animals
Source: PLoS One. 2015 Nov 4;10(11):e0142051. doi: 10.1371/journal.pone.0142051 (PMC4633224; doi:10.1371/journal.pone.0142051)
Supplement: S1 Table — (DOCX) [file pone.0142051.s001.docx]

Supplementary Table 1: Data for C-peptide Analysis

|  | Creatinine (mg/mL) | | | | Absolute C-peptide (ng/mL) | | | | C-peptide per Creatinine (ng/mg Cr) | | | |
| --- | --- | --- | --- | --- | --- | --- | --- | --- | --- | --- | --- | --- |
| Sample | Control | Synthetic | Cotton | First Aid | Control | Synthetic | Cotton | First Aid | Control | Synthetic | Cotton | First Aid |
| Human 1 | 0.77 | 0.81 | 0.67 | 0.70 | 53.2 | 39.6 | 59.8 | 67.6 | 69.1 | 48.9 | 89.3 | 96.6 |
| Human 2 | 0.45 | 0.50 | 0.44 | 0.38 | 41.8 | 27.8 | 41.8 | 16.4 | 92.9 | 55.6 | 95.0 | 43.2 |
| Human 3 | 3.65 | 3.65 | 3.15 | 3.45 | 82.4 | 83.8 | 131.8 | 124.2 | 22.6 | 23.0 | 41.8 | 36.0 |
| Human 4 | 4.55 | 4.35 | 3.50 | 4.05 | 111.6 | 111.2 | 112.4 | 130.4 | 24.5 | 25.6 | 32.1 | 32.2 |
| Rhesus 1 | 0.88 | 0.93 | 0.79 | 0.83 | 23.6 | 21.2 | 24.4 | 22.0 | 26.8 | 22.8 | 30.9 | 26.5 |
| Rhesus 2 | 0.89 | 0.92 | 0.81 | 0.84 | 22.3 | 18.7 | 23.0 | 18.9 | 25.1 | 20.3 | 28.4 | 22.5 |
| Rhesus 3 | 0.23 | 0.22 | 0.22 | 0.21 | 14.4 | 13.3 | 16.7 | 14.6 | 62.6 | 60.5 | 75.9 | 69.5 |
| Long-tailed | 1.12 | 1.22 | 0.96 | 1.05 | 18.0 | 19.0 | 20.6 | 21.4 | 16.1 | 15.6 | 21.5 | 20.4 |

|  |  |  |  |  |
| --- | --- | --- | --- | --- |
