# Supplementary material for: Validation of a Novel Collection Device for Non-Invasive Urine Sampling from Free-Ranging Animals
Source: PLoS One. 2015 Nov 4;10(11):e0142051. doi: 10.1371/journal.pone.0142051 (PMC4633224; doi:10.1371/journal.pone.0142051)
Supplement: S2 Table — (DOCX) [file pone.0142051.s002.docx]

Supplementary Table 2: Data for Neopterin Analysis

|  | Creatinine (mg/mL) | | Absolute Neopterin (ng/mL) | | Neopterin per Cr (ng/mg Cr) | |
| --- | --- | --- | --- | --- | --- | --- |
| Sample | Control | Synthetic | Control | Synthetic | Control | Synthetic |
| 1 | 2.55 | 2.70 | 520.0 | 420.0 | 203.9 | 155.6 |
| 2 | 0.90 | 0.77 | 244.0 | 214.0 | 268.9 | 277.9 |
| 3 | 2.85 | 3.10 | 360.0 | 310.0 | 126.3 | 100.0 |
| 4 | 0.74 | 0.74 | 80.0 | 76.0 | 106.7 | 102.7 |
| 5 | 1.21 | 1.27 | 98.0 | 96.0 | 82.4 | 75.6 |
| 6 | 0.21 | 0.23 | 14.0 | 15.0 | 60.9 | 65.3 |
| 7 | 0.27 | 0.28 | 36.0 | 36.0 | 135.7 | 128.6 |
| 8 | 0.41 | 0.42 | 32.0 | 38.0 | 78.0 | 90.5 |
| 9 | 0.18 | 0.19 | 17.0 | 15.0 | 94.1 | 78.9 |
| 10 | 0.33 | 0.35 | 74.0 | 82.0 | 236.4 | 234.3 |
| 11 | 0.70 | 0.66 | 144.0 | 150.0 | 205.7 | 227.3 |
| 12 | 0.67 | 0.72 | 126.0 | 146.0 | 182.6 | 202.8 |
